# Progress in Medicine

**Published:** 1897-02-27

**Authors:** 


### Progress in Medicine.

### FEVERS.

**Typhoid.**—A really important discovery seems to be due to Widal, who announced last summer that doubtful cases of typhoid could be diagnosed by the effect of a drop of their blood on the bacillus in a cultivation tube. Metchnikoff, Pfeiffer, and many others noted the action of serum from immune animals on specific bacilli, driving them into clumps, and depriving them of the power of motion when they possess it. Widal[1] now adds one drop of serum from the blood obtained by pricking the finger of a typhoid patient after sterilisation of the skin to ten drops of a recent cultivation of the bacillus. A drop is then examined under the microscope immediately and at intervals up to two hours. Serum obtained at the end of the first week or later causes this agglomeration. Serum from patients suffering from other diseases does not give the test, which fails also with the bacillus coli. Blister serum, tears, and milk cause the reaction, but urine is uncertain. Rodet[2] found that the serum of animals partly immunised against the typhoid or B. coli caused this reaction in their respective cultivations. Among various observers who confirm Widal's results, we may mention Pughesi,[3] Stern,[4] McWeeney, Coleman, Dempster,[5] and Delepine.[6] The latter describes minutely the technique of the method he has used. He found the reaction a doubtful test in only one case out of forty. He obtained results from patients after the first week of the disease, and most easily during the third.

Widal[7] noticed that the serum of convalescent patients gradually loses its power, and requires to be used in larger quantities, while during the fever one drop will produce the reaction in sixty to eighty drops of culture. Even a drop of dried blood, if moistened, suffices; and Dieulafoy[8] finds that the power is confined to the fibrinogen, globulin, and in milk to the casein. Grunbaum[9] claims to have arrived independently at the discovery of this reaction during fever as opposed to that of the serum of immunised animals, but insists that no change occurring after thirty minutes should be regarded. By another method of employing larger quantities of serum, it appears that the serum of many different diseases, or even that of health, will, unless diluted, cause the reaction. Thus Durham[10] prefers to take ·2 or ·3 cc. of blood by pricking the sterilised skin of the ear and drawing it into a pipette, which is emptied into a sterilised test tube. This is either centrifuged or the serum allowed to separate, and the latter is then diluted with 94 per cent. of water. To 30 cc. of this dilution ·5 cc. of a young bacillary culture is added. Even with dilution absolute certainty is not obtained in every case. Wyatt Johnston and McTaggart[11] advise that a drop of blood should be dried on sterilised non-absorbent paper, which can even be sent by post. It is then moistened with a drop of distilled water, and added to a drop of the culture on a coverslip. This is examined by a dry lens over a cell, or the slip is placed over a hole cut in blotting paper on a common slide. In a few minutes the bacilli run together in loose clumps. The reaction may sometimes be delayed for hours in undoubted cases, and, on the other hand, too concentrated serum may cause instantaneous stoppage of movement, followed by slow clumping. In this way they examined 290 cases, and obtained satisfactory results in from 95 to 99 per cent. Incomplete reactions were obtained as early as the second day of the disease, and complete generally about the sixth. A like reaction was obtained with cholera serum and cultures.

Special tubes and pipettes have been invented by A. E. Wright,[12] and in these clumping produces a cloud or precipitate visible to the naked eye, though only a drop or two of blood is used.

G. Bates Block[13] tested twenty cases in Professor Osler's wards with success, except as to three, where the reaction was slight. In two cases of diabetes and malaria the reaction by typhoid cultures was obtained, though some motility of the bacilli remained. C. L. Lyman Greene,[14] in speaking of the great accuracy of the test in his own cases, remarks that it can usually be observed with the naked eye. When an ordinary slide and cover glass are used a peculiar mottled appearance shows the clumping of the bacilli. Achard and Bensaude[15] found that clumping took place in cholera cultures with the serum of cholera patients even as early as the first day, and propose it as a test for the disease. Among other tests T. A. Codd[16] finds that Ehrlich's diazo reagent quickly decomposes, and recommends tabloids of sulphanilic acid 5-6ths grain to be dissolved with the other reagent in a hundred drops of urine and five drops of strong H.Cl. Afterwards a few drops of ammonia are poured in to neutralise it.

In typhoid, according to Chiari,[17] the bacilli can nearly always be found in the gall bladder, though it may be impossible to obtain a culture from the blood of the heart or the cerebrospinal fluid. Whether they enter the bladder by the ducts or through the blood is uncertain, but gall-stones and relapses are possibly a result of their presence. Bernheim[18] classifies the types of fever as including not only (*a*) the normal three-week course, but (*b*) that with shortening of the stationary period, or abortive typhoid, (*c*) that with prolongation of it, (*d*) that marked by recrudescence where a new evolution of bacilli takes place during a pyrexial period, (*e*) that marked by relapse where the new evolution occurs after convalescence has set in, (*f*) and that where secondary infection of streptococci and other germs takes place.

*Treatment.*—G. B. Henshaw[19] reports thirteen instances of the use of thymus extract, in eight of which satisfactory results were obtained. In four other cases the disease was not shortened, and one death from hæmorrhage occurred. He thinks that a stronger extract might be employed at an early date with advantage. Pfeiffer and Kolle[20] have investigated a method of typhoid prevention by inoculation of sterilised cultures of the bacillus. A c.cm. of a bouillon preparation sterilised at 56 deg. C. was injected into healthy individuals, and produced an attack of shivering vertigo and pyrexia, which disappeared in twenty-four hours. Certain changes in the blood serum occurred analogous to those found in typhoid convalescents, which lasted at least six days, and from the analogy of cholera they believe that immunity will be thus obtained. Ransom[21] reports that the mortality at the General Hospital, Nottingham, for five years was only 8·2 per cent., and that of the borough 15·5, and remarks on the danger from urine and sputum, and the dust of dried excreta.

M. E. Paul[22] notices that the percentage of abortive cases in patients who have had a previous attack is apparently unknown, but he mentions one which lasted nine days only, and was ushered in by an outbreak of herpes zoster. Duchenne[23] praises the treatment by copious draughts of cold water and other beverages except broth, and cold enemata; but in the discussion at the Therapeutic Society of Paris it was claimed that these cold enemata are absolutely inert, having neither antithermic nor diuretic properties. Another paper on this method, combined with calomel and salines and salol, is contributed by Thistle,[24] whose writings have excited much interest in America. He argues that a large part of the evil symptoms are due to secondary infections, and refers to the proved increase of virulence in the bacillus coli when Eberth's is present. Moreover, there is no proof that the typhoid bacilli are really absent from the intestine during the first nine days, which has been asserted; and, indeed, by the new method of Elsner they can be detected in the fæces, while the diagnosis is still doubtful. In any case, he reckons that 80 per cent. of typhoid mortality is due to toxin poisoning, and only 20 per cent. to accidental complications, such as hæmorrhage. Hence the importance of carrying away rapidly the poisons as they are formed. The cold-bath plan produced at the Presbyterian Hospital in New York a mortality of 7·25, or about that recorded in other American institutions; the calomel and guiacol treatment of Woodbridge was tested by Delafield,[25] who found some advantage from it only in mild cases, but certain cases seem to abort altogether at once if placed on calomel, as Waldstein and others noticed, though it is possible to assert here an error in diagnosis. Treatment by saline purgatives and especially by Rubinat water, given so as to produce four or five watery stools every other day, is preferred by C. E. Skinner.[26] He finds that strychnia is the best remedy against œdema of the lungs, and is in favour of a solid diet whenever the patient can digest it. The question of solid food has been lately revived in this country by A. G. Barrs, of Leeds, who has treated 28 cases without a death, and believes that the ulcers are hindered from healing by a prolonged milk diet in convalescence. In the acute stage, if a patient is hungry and wishes for solid food, such as meat, he gives it, but not otherwise. With regard to the danger of perforation, he believes, too, that this is lessened by copious nourishment, and that meat and bread become liquid before they reach the ulcerated tract, at least as much as milk curd; neither are they as likely to cause flatulent distension and stretching of the injured intestine. This remarkable theory has since been answered by Samuel O. West.

*Complications and Pathology.*—Gangrene has been reported in 203 cases, and, according to Keen,[27] in the Shattuck lecture, it may depend on arterial embolism or thrombosis, venous thrombosis, or peripheral thrombosis. The clots may be discontinuous, and the gangrene symmetrical. As to treatment, stimulation by good food, air, and alcohol, and the use of the Faradic current is recommended. Where operative measures are needed, the final line of demarcation must be waited for as a general rule, but if septic infection or speedy exhaustion appears, immediate amputation may be necessary at the probable limit. To determine this limit he analyses carefully the recorded cases. E. B. Steel[28] reports a case of perforation, peritonitis, and perityphlitic abscess from which four ounces of pus were evacuated. The patient made a good recovery. No less than six grains of opium in three hours were administered during the peritonitis. In peritonitis due to perforation it may be useful to remember Dieulafoy's[29] remark, that the constantly occurring fall of temperature does not disappear, like that from other causes, but remains for at least some days. Peritonitis may arise also, he thinks, from appendicitis occurring during the later stages of typhoid, which includes the so-called propagation peritonitis, where it is usually said the inflammation has passed through the wall of the intestine. It may be remembered that Schultz and others recently asserted that the leucocytes are heaped up in certain parts of the circulation in typhoid, and that the cold bath restores their even distribution. Semakine[30] denies the evidence, and tries to show that in life the corpuscles are equally distributed in the central and peripheral vessels.

The improved tests for the typhoid bacillus are already bearing fruit in various quarters. Remheifer and Sneider[31] were able to isolate this microbe in earth, in water from various places, and in the stools of certain individuals not suffering from typhoid. They employed Elsner's medium for isolating the bacillus, and found that in many instances the bacillus thus obtained was pathogenic for animals, unless previously rendered immune by typhoid inoculations. This, of course, formed a second test of its identity. The oyster scare is the cause of a report of the Local Government Board recently issued. Laboratory tests seem from it to be of little value for the discrimination of contaminated from healthy molluscs, but regulations are needed to prevent oysters being laid down or stored in polluted waters. Moreover, it is shown that the typhoid bacillus can live at least two weeks in sea water.

The micro-organism of typhus is described by Levaschew[33] as a minute coccus grouped in various ways, sometimes possessing a single flagellum, but often motionless, staining in fuchsin or methylene blue and decolourised by alcohol. Those obtained from venous blood grow on ordinary media at 36 deg. or 37 deg (provided they are not acid or saccharine). It is äerobic, and was found in 156 out of 158 cases of typhus. Several other observers have noted organisms, which are probably identical with this. At Jerusalem a continued fever is very prevalent, and is often confounded with typhoid. Friess[34] believes it to be really malarial, though it last several weeks, and resists quinine and all other remedies. There is high pyrexia, few complications, and a very small mortality. The bowels are generally constipated, there is no gurgling in the right iliac forsa and recovery is rapid. On the whole the affection is not unlike the special fevers of Malta and Athens.

[1] Med. Chronic., Oct. [2] N. Y. Med. Jour., Oct. 10. [3] B. Med. Jour., Jan. 2. [4] Centralblatt f. In. Med., Dec. 5. [5] B. M. J., Jan. 16. [6] Lancet, Dec. 5. [7] Med. Week, Oct. 16. [8] Med. Week, Oct. 2. [9] Lancet, Dec. 19. [10] Ibidem. [11] B. Med. Jour., Dec. 5. [12] B. Med. Jour., Jan. 16. [13] Johns Hopkins Bulletin, No. 68, 69. [14] N. Y. Med. Jour., Dec. 5. [15] Presse Med., Sept. 26. [16] Birmingham Med. Rev., Nov. [17] B. Med. Jour., Aug. 29. [18] Med. Week, Sept. 4. [19] Am. J. Med. Sciences, Sept. [20] B. Med. Jour., Jan. 2. [21] B. M. Jour., Dec. 12. [22] Lancet, Dec. 26. [23] Med. Week, Oct. 23. [24] Med. Rec., Oct. 17. [25] Med. Rec., Nov. 7. [26] N. Y. Med. Jour., Oct. 24. [27] Therap. Gazette, Nov. 16. [28] B. Med. Jour., Jan. 2. [29] Med. Week, Oct. 30. [30] Lancet, Dec. 5. [31] Amer. Jour. Med. Sciences, Oct. [32] B. Med. Jour., Dec. 5. [33] B. Med. Jour., Dec. [34] N. Y. Med. Jour., Aug. 15.

